# Experimental Adaptation of Rotaviruses to Tumor Cell Lines

**DOI:** 10.1371/journal.pone.0147666

**Published:** 2016-02-01

**Authors:** Carlos A. Guerrero, Rafael A. Guerrero, Elver Silva, Orlando Acosta, Emiliano Barreto

**Affiliations:** 1 Department of Physiological Sciences, Faculty of Medicine, Universidad Nacional de Colombia, Bogota, D.C., Colombia; 2 Institute of Biotechnology, Universidad Nacional de Colombia, Bogota, D.C., Colombia; University of Saarland Medical School, GERMANY

## Abstract

A number of viruses show a naturally extended tropism for tumor cells whereas other viruses have been genetically modified or adapted to infect tumor cells. Oncolytic viruses have become a promising tool for treating some cancers by inducing cell lysis or immune response to tumor cells. In the present work, rotavirus strains TRF-41 (G5) (porcine), RRV (G3) (simian), UK (G6-P5) (bovine), Ym (G11-P9) (porcine), ECwt (murine), Wa (G1-P8), Wi61 (G9) and M69 (G8) (human), and five wild-type human rotavirus isolates were passaged multiple times in different human tumor cell lines and then combined in five different ways before additional multiple passages in tumor cell lines. Cell death caused by the tumor cell-adapted isolates was characterized using Hoechst, propidium iodide, 7-AAD, Annexin V, TUNEL, and anti-poly-(ADP ribose) polymerase (PARP) and -phospho-histone H2A.X antibodies. Multiple passages of the combined rotaviruses in tumor cell lines led to a successful infection of these cells, suggesting a gain-of-function by the acquisition of greater infectious capacity as compared with that of the parental rotaviruses. The electropherotype profiles suggest that unique tumor cell-adapted isolates were derived from reassortment of parental rotaviruses. Infection produced by such rotavirus isolates induced chromatin modifications compatible with apoptotic cell death.

## Introduction

Although a small decrease in the overall cancer rate has been reported for countries such as USA, the incidence of some particular cancers has shown an increasing rate [1]. Scientists worldwide are constantly searching for new therapies for treating cancer other than the conventional chemotherapy or radiotherapy [2,3]. Viruses exhibit cellular tropism which defines their ability to preferentially infect a specific tissue. A number of viruses have been shown to naturally extend their tropism to tumor cells [4]. Reovirus, parvovirus, Newcastle disease virus (NDV), Moloney leukemia virus (MLV) and mumps virus (MV) are among the viruses showing natural preference for tumor cells, while viruses such as vesicular stomatitis virus (VSV), measles virus (MV), vaccinia virus (VV), adenovirus (AdV), and herpes simplex virus (HSV) have been genetically modified or adapted to infect tumor cells [5]. Oncolytic viruses have recently become a promising tool for treating cancer by producing lysis of tumor cells or inducing an immune response to them [4]. Some desirable characteristics of viruses can be modified by redesigning their genome in order to improve viral tropism to neoplastic cells, enhance lytic capacity or induce antitumor immunity [5]. Strategies targeting tumor vasculature have also involved oncolytic viruses [6]. Genetically-modified adenoviruses showing oncolytic and antiangiogenic properties have been combined to synergistically increase antitumor effect [7]. Entry of some oncolytic virus is mediated by specific or over-expressed receptors on the tumor cell surface [8]. These viruses use the molecular cell death machinery of the tumor cell in order to ensure their replication and assembly before the occurrence of cell death [9]. The viral oncolytic approach has been demonstrated in many preclinical cancer systems and in early and late phase clinical trials including solid and hematological cancers [10–13].

Rotavirus, a member of the family *Reoviridae*, has a triple-layered protein capsid that contains 11 doubled-stranded RNA genomic segments and infects mature enterocytes from human and many animal species [14]. Many rotaviruses have been adapted to grow in cultured human and animal cell lines [15]. Rotavirus infection requires the presence of some cell surface receptors such as PDI [16], Hsc70 [17] and integrins, including αVβ3 [18]. These cell surface molecules have been shown to be associated in lipid microdomains (rafts) facilitating rotavirus entry [19,20]. Rotaviruses infect the non-dividing mature enterocytes localized near the tips of small intestinal villi causing alterations that lead to severe and dehydrating diarrhea [21]. Interestingly, rotavirus tropism is determined by the presence of receptor molecules that are properly located on the enterocyte cell surface but absent, reduced or differently associated in cells other than enterocytes [22,23]. Natural rotavirus tropism specificity mediated by cell surface receptors has been demonstrated by infecting rotavirus resistant cells using infectious triple layered particles (TLPs) or non-infectious double layered particles (DLPs) contained in liposomes that through fusion with cell membrane circumvent the need for cell surface receptors [24–26]. This remarkable property of the natural history of rotaviruses makes them promising tools for being used as oncolytic agents. Cell malignancy has been associated with expression of PDI [27–29], Hsc70 [30–35], integrin β3 [36–39], and several heat shock proteins (HSPs) [33–35]. PDI [40] and Hsc70 [41] are often expressed at very low levels or not at all on the cell surface of normal cells, whereas neoplastic cells do express them, and several HSPs, at relatively high levels [32,42,43]. We adapted some rotaviruses to grow in tumoral cell lines that are representative of the most epidemiologically important human cancers. In the present study, we present evidence of the adaptation of five rotavirus isolates that were able to replicate and induce cell death in some tumoral cell lines. To our knowledge, this is the first study attempting to adapt rotaviruses to tumor cell lines with oncolytic purposes.

## Materials and Methods

### Cells

MA104 cells (African green monkey kidney, ATCC^®^ CRL-2378.1) and Caco-2 cells (human colon adenocarcinoma, ATCC^®^ HTB-37) were obtained from Dr. C. F. Arias (Instituto de Biotecnología, Universidad Nacional Autónoma de Mexico). Peripheral blood mononuclear cells (PBMC) were isolated using Ficoll-Paque^™^. AGS cells (human gastric adenocarcinoma, ATCC^®^ CRL-1739), Kato III cells (human gastric carcinoma, ATCC^®^ HTB-103), MCF-7 cells (human breast adenocarcinoma cell, ATCC^®^ HTB.22), PC-3 cells (human prostate adenocarcinoma, ATCC^®^ CRL-1435), U937 cells (human promyelomonocytic leukemic, ATCC^®^ CRL-1593.2), and L929 cells (L cell, L-929, derivative of Strain L, ATCC^®^ CCL-1^™^) were kindly donated by Dr. C. Parra, Faculty of Medicine, Universidad Nacional de Colombia). REH cells (human acute lymphocytic leukemia—non-T; non-B, ATCC^®^ CRL-8286^™^) were kindly donated by Dr. J. P. Vernot, Faculty of Medicine, Universidad Nacional de Colombia). A549 cells (human lung carcinoma, ATCC ^®^ CRL-185), and Sp2/0-Ag14 cells (mouse B cell myeloma, ATCC^®^ CRL-1581) were obtained from ATCC^®^. All cell lines were cultured in Dulbecco´s Modified Eagle Medium (DMEM) or RPMI 1640 (Sigma-Aldrich, St. Louis, MO, USA) supplemented with 10% fetal bovine serum (FBS) (Eurobio, Les Ulis, France) and 100 μg/ml streptomycin and penicillin (Eurobio, Les Ulis, France). All cells were cultured in a humidified atmosphere with 5% CO2 at 37°C.

### Parental rotaviruses

Rotavirus strains TRF-41 (G5) (porcine), RRV (G3) (simian), UK (G6-P5) (bovine), Ym (G11-P9) (porcine), Wa (G1-P8), Wi61 (G9) and M69 (G8) (human) were kindly provided by Dr. C. F. Arias, Instituto de Biotecnología, Universidad Nacional Autónoma de Mexico, and propagated in MA104 cells. Rotavirus strain ECwt (wild-type murine rotavirus EDIM-Cambridge) was kindly provided by Dr. M. Franco, Universidad Javeriana, Bogotá, and propagated in ICR suckling mice. Wild type rotaviruses (WT1, WT2, WT3, WT4 and WT5) were purified from five children.

### Generation of new isolates

Parental rotavirus strains previously activated with trypsin (10 μg/ml) for 20 min at 37°C were passaged in human cell line cultures U937, AGS, Kato III, MCF-7, PC-3, REH and A549, and murine cell line Sp2/0-Ag14. Before virus inoculation, the culture medium was removed and cells were washed twice with MEM. Cells (1 x 10^7^) contained in 5 ml were inoculated with 500 μl of a virus preparation of unknown titer. After inoculation, lysis of cells was typically observed between 48 and 72 hours post-infection (h.p.i.) during the first 50 passages. Following further passages, the lysis period decreased to 24–48 h.p.i. Cell lysates were centrifuged at 14000 *g* for 10 min and the supernatant was used for the next passages. A 1:10 dilution of supernatant was carried out only whether the cell lysis had occurred before 48 h.p.i. After at least 150 passages for each parental virus in each tumor cell line, the resultant tumor cell-passaged virus preparations were pooled. The infectious capacity of the supernatants from these pooled preparations was compared to that of the corresponding parental inoculum. The infectious titer for each parental rotavirus and its corresponding tumor cell-passaged virus preparation was determined in MA104 cells. The infectious titer was expressed as focus forming units per milliliter (FFU/ml). These infectious titers were compared with those determined in the cell lines AGS, U937, Sp2/0-Ag14, MCF-7, and Caco-2. Virus titer for both parental and tumor cell-passaged virus preparations was determined on cells (5 × 10^4^/well) grown in adherent or suspension cultures.

Four different combinations of viruses were created by mixing equal infectious titers of several different tumor cell-passaged rotaviruses that had been pooled after 150 passages: 1. Rotavirus TRF, RRV, UK, and Ym; 2. Rotavirus Wa, Wi, and M69; 3. Five rotaviruses (WT1, WT2, WT3, WT4 and WT5) purified from children as previously described [44] [45]. These combinations were named as TRUY, WWM and WT1-5, respectively. To facilitate the emergence of new rotavirus variants, each combination was passaged at least 100 times in each cell line (U937, AGS, Kato III, MCF-7, PC-3, REH, A549, and Sp2/0-Ag14). 4. After the three different rotavirus combinations (TRUY, WWM and WT1-5) were passaged as indicated above, they were pooled and mixed together with the pool of tumor cell-passaged ECwt preparations to generate the combination WTEW, which was subjected to 100 passages in each cell line (U937, AGS, Kato III, MCF-7, PC-3, REH, A549 and Sp2/0-Ag14). After all virus combinations were subjected to 100 passages in each cell line, the resultant virus preparations were pooled and henceforth named as tumor cell-adapted isolates.

### Infection of cells

Culture medium was removed from cells (AGS, U937, Sp2/0-Ag14, MCF-7, REH, Caco-2, MA104, PBMCs or L929) before washing them twice with MEM. About 5 × 10^4^ cells per well in 50 μl of MEM without FBS were inoculated with trypsin-activated viral preparations and incubated in 96-well plastic culture plates at 37°C for 12 h in a 5% CO_2_ incubator. To compare the infectivity of parental rotavirus strains/isolates with that of each rotavirus isolate adapted to tumor cell lines (AGS, U937, Sp2/0-Ag14, MCF-7 and Caco-2), MA104 cells infected with parental rotavirus strains/isolates or tumor cells lines infected with tumor cell-adapted rotavirus isolates were harvested at 12 h.p.i. Lysates from infected cells were serially diluted in MEM and titered in MA104 cells. Infectivity comparisons were performed by inoculating MA104 cells and the respective tumor cell lines with either parental rotavirus strains/isolates or tumor cell-adapted rotavirus isolates at MOI of 0.02 as determined in MA104 cells. The percentage of rotavirus antigen-positive cells was determined at 12 h.p.i. by fixing the cells in ice-cold methanol before being subjected to immunochemistry assay.

The cytopathic effect produced by isolates WT1-5, TRUY, WWM, WTEW or ECwt on tumor cell lines was analyzed by infecting the cells with 0.8 MOI of each trypsin-activated isolate as determined in the respective tumor cell line. Adherent or suspension cells were harvested at 12 h.p.i. to assess the infectious titer of their viral yield as indicated above. To determine the cytopathic effect, the cells were harvested every 2 hours until 12 h.p.i., and a cell aliquot was also harvested at 24 h.p.i. Cells were fixed with 4% paraformaldehyde (PFA) in PBS for 30 min at room temperature (RT). Afterwards, cells were washed twice with PBS and resuspended in PBS containing 0.02% sodium azide before being stored at 4°C until use. For adherent cells, MA104 and L929 monolayers were washed with MEM before inoculation with trypsin-activated viral isolates in serial dilutions. Cells were harvested at 12 h.p.i. and fixed with ice-cold methanol for 1 h. The percentage of infected cells in terms of rotavirus antigen-positive cells was assessed by immunocytochemistry.

To determine the production of infectious virions by tumor cells Sp2/0-Ag14, U937 and REH infected with the tumor cell-adapted rotavirus isolates WT1-5, TRUY, WWM, WTEW or ECwt (MOI of 0.8), the infected cells were harvested every 2 h until 12 h.p.i. and lysed by two cycles of freezing and thawing. Infectious titers of cell lysates were determined in the respective cell line using immunocytochemistry assay. The production of infectious virions was further examined by infecting the tumor cells Sp2/0-Ag14, U937 and REH with the tumor cell-adapted virus indicated immediately above. The cells harvested every 2 h until 12 h.p.i. were frozen and thawed twice, and centrifuged at 600 *g* for 10 min. The supernatant from each postinfection time point was activated with trypsin and tested at serial dilutions in new Sp2/0-Ag14, U937 and REH cells. The infected cells were fixed at 12 h.p.i. and the corresponding infectious titer determined by immunocytochemistry.

To test for the release of infectious virions to the medium, the tumor cells Sp2/0-Ag14, U937 and REH were infected with the tumor cell-adapted rotavirus isolates WT1-5, TRUY, WWM, WTEW or ECwt (MOI of 0.8) and cells harvested every 2 h until 12 h.p.i. The cell suspension was centrifuged at 700 *g* and the supernatant collected to be analyzed for the presence of viral antigens using ELISA as indicated below.

### Immunocytochemistry

Suspension cells were fixed in 4% PFA for 30 min at RT, placed onto glass slides previously cleaned with xylol and then treated with ice-cold methanol. Cells were dried for approximately 30 min in an oven at 50°C. After permeabilization in 0.5% Triton X-100 solution for 5 min at RT, the cells were washed twice in PBS and incubated with primary rabbit polyclonal antibodies (Abs) (produced in our animal facilities) against rotavirus structural proteins (SP) or non-structural proteins (NSP4 and NSP5) for 1 h at 37°C. After washing twice with PBS, the cells were incubated with secondary HRP-conjugated goat anti-rabbit Abs (0.133 μg/ml, Santa Cruz Biotechnology Inc., Santa Cruz, CA, USA) for 1 h at 37°C. Following several washes with PBS, cells being positive to rotavirus antigen were visualized with 0.64 mg/ml of 3-amino-9-ethylcarbazole (AEC) substrate in 50 mM acetate buffer, pH5-0, containing 0.04% H_2_O_2_. Non-infected tumor cells or infected tumor cells treated with non-related antibodies were used as a control. At least ten representative images were photographically recorded using a conventional light microscope (VanGuard, Scottsdale, USA) equipped with a camera, and the mean percentage of infected cells was determined.

### Fluorescence analysis

To assess the expression levels of heat shock proteins (HSPs) (Hsp90, Hsp70, Hsp60 and Hsp40), Hsc70, COX-2, NF-*κ*B and PDI, cells were infected with the individual rotavirus isolates WT1-5, TRUY, WWM, WTEW or ECwt at a MOI of 0.8 as indicated above for the immunocytochemistry assay. Infected cells were fixed with ice-cold methanol for 45 min and then placed onto coverslips. After drying, cells were permeabilized and treated with a solution of 50 mM ammonium chloride and 100 mM glycine in PBS for 30 min at RT. Afterwards, cells were simultaneously incubated for 1 h at 37°C with a mix of Abs consisting of mouse mAbs against HSPs (90, 70, 60 and 40), goat polyclonal Abs against Hsc70, PDI, COX-2 or NF-*κ*B (0.2 μg/ml, Santa Cruz Biotechnology Inc., Santa Cruz, CA, USA) and rabbit polyclonal antibodies against rotavirus SP, NSP4 and NSP5 in PBS containing 1% BSA. Following two washes with PBS, the cells were incubated for 30 min at 4°C in darkness with Alexa-568-conjugated donkey-anti-goat or goat-anti-mouse or FITC-conjugated chicken-anti-rabbit secondary Abs (5.7 μg/ml, Invitrogen, Carlsbad, CA, USA) in PBS containing 1% BSA. The coverslips were mounted (inverted) on glass slides using 70% glycerol in PBS and resin, and examined by confocal microscopy. Uninfected cells were used as a control.

To assess the cytotoxic effect of the rotavirus infection on the tumor cell lines, cells were separately infected with the individual rotavirus isolates WT1-5, TRUY, WWM, WTEW or ECwt at a MOI of 0.8. The cells were harvested at 0 and 12 h.p.i. and washed twice with PBS. Cells (1 × 10^4^) were resuspended in 50 μl of HEPES buffer, pH 7.4, containing 10 mM NaCl, 5 mM CaCl_2_ and Annexin-V-Alexa 568 (20 μl/ml) (Roche, Indianapolis, IN, USA) and kept for 15 min at RT. Cells treated with 1 mM H_2_O_2_ for 12 h were used as control.

Cell death of tumor cell lines infected with rotavirus isolates were estimated using 7-AAD staining (Invitrogen, Carlsbad, CA, USA). Tumor cells were infected with the different rotavirus isolates at a MOI of 0.8 and collected at 0 and 12 h.p.i. After washing twice with PBS, 1 × 10^4^ cells were collected by centrifugation (600 *g*) and then resuspended in 1 ml of 7-AAD (5 μg/ml) in PBS containing 300 mM CaCl2, MgCl2, 0.2% BSA and 0.1% sodium azide. After incubation for 20 min at 4°C in darkness, the cells were washed twice with PBS and analyzed using a fluorescence microscope. Rotavirus infected and non-infected tumor cells were used as a control after treatment with 1 mM H_2_O_2_.

DNA damage induced by rotavirus infection was detected with a commercially available kit using an Ab against poly-(ADP ribose) polymerase (PARP) (Roche, Indianapolis, IN). The procedure was conducted according to the manufacturer instructions. Briefly, cells were fixed with 4% PFA, placed onto coverslips previously cleaned with xylol, and then allowed to dry for about 30 min in an oven at 50°C. The cells were then permeabilized in 0.5% Triton X-100 for 5 min at RT. After washing twice with PBS containing 1% BSA and 1% Tween 20, the cells were incubated with a rabbit anti-PARP polyclonal Ab (0.5 μg/ml) in PBS solution for 1 h at RT. After washing twice with PBS, secondary FITC-conjugated goat anti-rabbit IgG (0.66 μg/ml, Santa Cruz Biotechnology Inc., Santa Cruz, CA, USA) was added and incubated for 30 min at RT. The cells were washed twice with PBS and then mounted onto glass slides with 70% glycerin and sealed with resin.

Damage of DNA was also assessed by detecting the phosphorylation of histone H2AX that occurs at Ser 139. Cells that had been separately infected (MOI of 0.8) with rotavirus isolates were harvested at 12 h.p.i. before being fixed as described for PARP assay. The cells were washed with PBS and incubated with anti-phospho-histone H2A.X (Ser 139) mouse monoclonal antibody (mAb) (Millipore, Darmstadt, Germany) overnight at 4°C. Incubation with FITC-conjugated donkey anti-mouse IgG (0.66 μg/ml, Santa Cruz Biotechnology Inc., Santa Cruz, CA, USA) was conducted for 30 min at RT. The cells were mounted on glass slides with 70% glycerin and sealed with resin.

Tumor cells were infected as described above for the different rotavirus isolates. After inoculation, the cells were harvested every 2 h until 12 h.p.i. or at 36 h.p.i. and fixed in 4% PFA. Following washing twice with PBS, 1 × 10^6^ cells were resuspended in 100 μl of PBS containing 0.02% sodium azide and stained with Hoechst 33342 (25 μl/ml, Thermo scientific Inc., Rockford, IL, USA) or propidium iodide (20 μg/ml) (Invitrogen, Carlsbad, CA, USA) for 10 min at RT. After washing twice with PBS, coverslips with the stained cells were mounted with 70% glycerol onto glass slides and sealed with resin.

In all cases, the fluorescence images were taken using a Nikon C1 Eclipse confocal laser microscope equipped with the Confocal Acquisition Software Nikon EZ-C1 version 3.90. DAPI staining was visualized using an exciting laser bean at 408 nm and a photodetector at 450/35 nm. Alexa 568 fluorescence was excited with a laser beam at 543 nm and recorded with a photodetector at 605/75. Photographs were taken at 100× magnification and the images analyzed using the software ImageJ 1.44 Java 1.6.0_20 de 32-bit.

### Cell viability

To determine cell viability the Trypan blue exclusion test was used. MA104 or L929 cell monolayers were treated with a 0.25% Trypan blue solution and incubated for 1 min at RT. After washing with PBS, cell viability was assessed using an inverted microscope (Euromex) at 40× magnification. In the case of tumor cells, suspensions of cells were mixed 1:1 (vol/vol) with a 0.25% Trypan blue solution and incubated as indicated for adherent cells, except that cell were observed in a Neubauer chamber using 40χ magnification.

### DNA fragmentation assay

Cells (1 × 10^7^/ml) were separately infected with the different rotavirus isolates at a MOI of 2. After12 h.p.i., the cells were harvested, added with 0.5 mM PMSF and kept at– 20°C. The DNA fragmentation was assessed by agarose gel electrophoresis. The apoptotic effect was determined with the Apoptotic DNA Ladder kit (Roche, Indianapolis, IN, USA). Cells (2 × 10^6^) in a volume of 200 μl of PBS were added with 200 μl of lysis buffer (6 M guanidine-HCl, 10 mM Urea, 10 mM Tris-HCl, pH 4.4, and 20% Triton X-100) and incubated for 10 min at RT. After addition of 100 μl of isopropanol, the preparation was stirred in a vortex for 1 min. By combining the filter and collection tubes provided in the kit, the samples were applied into the upper reservoir and centrifuged at 6200 *g* for 1 min. The eluted fraction was discarded and the filter and collector tubes were combined again. After adding 500 μl of washing buffer (mM NaCl; 2 mM Tris-HCl; 80% [v/v] ethanol: pH 7.5) the tubes were centrifuged at 2200 *g* for 1 min. The collection tube was discarded and the residual washing buffer was removed by centrifugation at 17000 *g* for 10 sec and applied into the upper reservoir. The filter tube was placed into an Eppendorf tube and the DNA eluted with 200 μl of 72°C pre-warmed elution buffer (10 mM Tris, pH 8.5) and centrifugation at 6200 *g* for 1 min. The procedure was performed twice and the eluted DNA stored at– 20 μl for subsequent use. Concentration and purity of DNA were determined using a NanoDrop 2000c (Thermo Scientific Inc., Waltham, MA, USA). DNA extracted from non-infected cells or cells treated with 1 mM H_2_O_2_ during 12 h were used as a control. DNA at a concentration of 100 μg/ml in loading buffer (40 mM Tris-acetate, pH 8.3, 1 mM EDTA, 0.5% SDS, 0.05% bromophenol blue, 40% sucrose). The DNA sample was analysed in a 1% agarose gel for 1.5 h at 75 V (5 V/cm) and the gel stained with ethidium bromide (0.5 μg/ml) and visualized under UV light.

DNA integrity was also assessed by in situ nick end labeling of programmed cell death-associated nuclear DNA fragmentation using a commercially available TUNEL kit (Invitrogen, Carlsbad, CA, USA). Cells (1.5 × 10^6^) in 1 ml of culture medium were placed into 12-well culture plates and then separately infected with the trypsin-activated rotavirus isolates WT1-5, TRUY, WWM, WTEW or ECwt at a MOI of 0.8. The cells were harvested after incubation for 12 h at 37°C. Viral infection was determined by immunocytochemistry as described above. After washing twice with PBS, the cells were placed onto coverslips and air-dried at RT to be subjected to analysis with the TUNEL kit. Non-infected cells and cells treated with 1 mM H_2_O_2_ for 12 h were used as a control. The cells were washed with PBS and the fluorescence due to the incorporated fluorescein-dUTP at DNA breaks was analyzed with a fluorescence microscope. At least ten representative photographs from each coverslip were analyzed. The positive fluorescence signals from infected and non-infected cells were established.

### Analysis of viral dsRNAs and proteins

Cell lines were separately infected with parental rotavirus strains/isolates or cell tumor-adapted rotavirus isolates WT1-5, TRUY, WWM, WTEW or ECwt at a MOI of 0.8 for 24 h at 37°C. Cell lysates (2.5 ml) or viral purified preparations (50 μl) were added with one-tenth volume of 10X lysis buffer (260 mM Tris-HCl, pH 8, 90 mM EDTA, 1.3% SDS, 680 mM NaCl and 0.1% 2-ME) and then extracted with phenol-chloroform (1:1). The RNA in the aqueous phase was precipitated with 2 volumes of ethanol at– 70°C overnight, collected by centrifugation at 14000 *g* for 10 min and suspended in 20 μl of loading buffer (62 mM Tris, pH 6.8, 2% (w/v) SDS, 0.001% (w/v) bromophenol blue and 10% (v/v) glycerol). Eletropherotyping of extracted dsRNA samples was performed by SDS-PAGE (8%) using the Laemmli buffer system and silver staining. Proteins remaining in the interface and organic phase were collected and precipitated with 5 volumes of acetone at– 20°C for 1h. The pellet was recovered by centrifugation at 14000 *g* and washed twice with cold 80% ethanol. Proteins were resolved by SDS-PAGE in a 10% gel and then transferred to a PVDF membrane. Viral proteins were detected with antibodies against rotavirus structural and non-structural proteins.

### ELISA

Capture ELISA was conducted as previously described [44]. Briefly, tumor cells were separately infected with the rotavirus isolates WT1-5, TRUY, WWM, WTEW or ECwt at a MOI of 0.8 and incubated for 12 h at 37°C. The cell suspension was centrifuged at 700 *g* and the supernatant collected to be analyzed for the presence of viral antigens. The supernatant was treated with 1/10 volume of 10X RIPA buffer (1X: 50 mM Tris-HCl, pH 8.0, 150 mM NaCl, 1% NP-40, 0.5% DOC, 0.1% SDS) followed by centrifugation at 2200 *g* for 10 min. The supernatant was applied to an ELISA plate coated with polyclonal guinea pig Abs (1:1000) against rotavirus particles. After incubation for 1 h at 37°C, the plate was washed with PBS and incubated with polyclonal rabbit Abs (1:3000) to rotavirus particles for 1 h at 37°C. After three washes with PBS, the plate was incubated for 1 h at 37°C with secondary HRP-conjugated goat anti-rabbit Abs (0.08 μg/ml, SC-2313, Santa Cruz Biotechnology, Inc., Santa Cruz, CA, USA). The reaction was visualized using o-phenilenediamine dihydrochloride (OPD) in stable peroxide substrate buffer and absorbance was read at 492 nm. Purified ECwt and supernatant from non-infected cells were used as a control. Mean background values from controls were subtracted from al data.

### Infection of peripheral blood mononuclear cells (PBMCs)

Blood (20 ml) was drawn from healthy volunteers using heparinized syringes and diluted by addition of 1 volume of RPMI 1640 before overlaying onto Ficoll-Hypaque (3:1 ratio) and centrifugation at 250 *g* for 30 min. The white PBMCs were collected from the interface and washed twice with FBS-free RPMI 1640 prior centrifugation at 400 *g* for 5 min. About 5 × 10^4^ cells in 200 μl of FBS-free RPMI 1640 were seeded in 24-well culture plates. A parallel culture of U937 cells was included. The cells were infected with the rotavirus isolates WT1-5, TRUY, WWM, WTEW or ECwt with a serial dilution of virus inoculum (MOI = 2, 1, 0.5, 0.25, 0.125, 0.062, 0.03, 0.015) and incubated for 12 h at 37°C. The infection was determined by immunochemistry using polyclonal rabbit Abs (1:2000) against rotavirus structural proteins and HRP-conjugated goat anti-rabbit secondary antibodies (0.13 μg/ml, Santa Cruz Biotechnology, Inc., Santa Cruz, CA, USA). At least ten representative photographs were taken and the mean percentage of infected cells was determined.

### Statistical analysis

The data that complied with the Levene's test of homogeneity of variance were subjected to one-way analysis of variance (ANOVA). Data are expressed as mean ± S.D. from at least two independent experiments. The non-paired Student’s t-test was used to compare means between infected and non-infected cells.

## Results

### Infectivity of isolates obtained after multiple passages of parental rotaviruses

To determine the effect of multiple cell culture passages on the infectivity of parental rotavirus strains (TRF-41, RRV, UK, Ym, Wa, Wi61, M69, WT1, WT2, WT3, WT4, WT5 and ECwt), cell lines U937, AGS, Caco-2, Kato III, MCF-7, PC-3, REH, A549, and Sp2/0-Ag14 were separately infected with these parental strains. After at least 150 passages, the infectivity of the parental strains was compared with that of the corresponding final isolates on the same cell lines used for culture passages. The comparison was made by inoculating serial dilutions of the parental strains or their corresponding tumor cell-adapted isolates on MA104 cells in order to obtain the same MOI (0.02) for all viral inocula before inoculation of the tumor cells used for adaptation of the isolates. When the differential infectivity was measured in terms of the percentage of infected cells, the results showed that most tumor cell-passaged rotavirus were able to infect a higher proportion of tumor cells AGS, U937, Sp2/0-Ag14 and MCF-7 and Caco-2 than MA104 cells. In contrast, most parental rotavirus strains exhibited less infectious capacity in these cells lines than in MA104 cells. These results indicate that every tumor cell-adapted isolate was significantly more infectious in every tumor cell line than its corresponding parental strains (Table 1). Adaptation conferred a relative infectivity increase that ranged from 1.3–28-fold greater than that found for the parental strains (Table 1). It is worthy to highlight the large variability of the infectious capacity of each parental rotavirus strain or tumor cell-adapted isolate when they infected the different tumor cell lines assayed. In other words, a broad susceptibility to viral infection was observed when the different tumor cell lines were infected with each parental rotavirus strain or isolate. Overall, the results suggest that all the adapted rotavirus isolates gained a relative infectious capacity to infect the tumor cells in which they were adapted in comparison to their parental versions.

**Table 1:** Infectivity of tumor cell-adapted rotavirus isolates compared with the infectivity of their respective parental versions.

Cell line	AGS			U937			Sp2/O-Ag14			MCF-7			Caco-2		
Virus	A^a^	B^b^	B/A	A	B	B/A	A	B	B/A	A	B	B/A	A	B	B/A
**WT1**	2	41	20.5	8	50	6.3	6	36	6	9	46	5.1	6	10	1.7
**WT2**	3	24	8	4	54	13.5	5	39	7.8	11	92	8.4	4	7	1.8
**WT3**	1	28	28	5	48	9.6	8	32	4	13	93	7.2	7	9	1.3
**WT4**	3	36	12	7	42	6	4	30	7.5	10	78	7.8	5	11	2.2
**WT5**	2	32	16	6	46	7.7	7	33	4.7	15	71	4.7	8	15	1.9
**Wa**	3	18	6	3	18	6	20	39	2	30	75	2.5	1.5	4	2.7
**69M**	9	30	3.3	7	39	5.6	13	45	3.5	25	80	3.2	1.4	3.5	2.5
**Wi**	2	7	3.5	17	32	1.9	18	43	2.4	23	70	3	1.6	4.1	2.6
**TRF**	7	46	6.6	14	36	2.6	10	37	3.7	20	84	4.2	1.6	3.9	2.4
**RRV**	9	39	4.3	2	34	17	12	36	3	40	80	2	1.4	3.8	2.7
**UK**	2	8	4	11	16	1.5	14	41	2.9	5	48	9.6	1.5	5	3.3
**Ym**	4	27	6.8	12	34	2.8	10	32	3.2	30	79	2.6	2.1	8	3.8
**ECwt**	6	20	3.3	5	12	2.4	16	59	3.7	16	36	2.3	1.4	4.7	3.4

^a^Infectivity (%) of parental strains/isolates

^b^Infectivity (%) of tumor cell-adapted isolates.

### Analysis of the rotavirus infection in tumor cell lines

**Fig 1:**
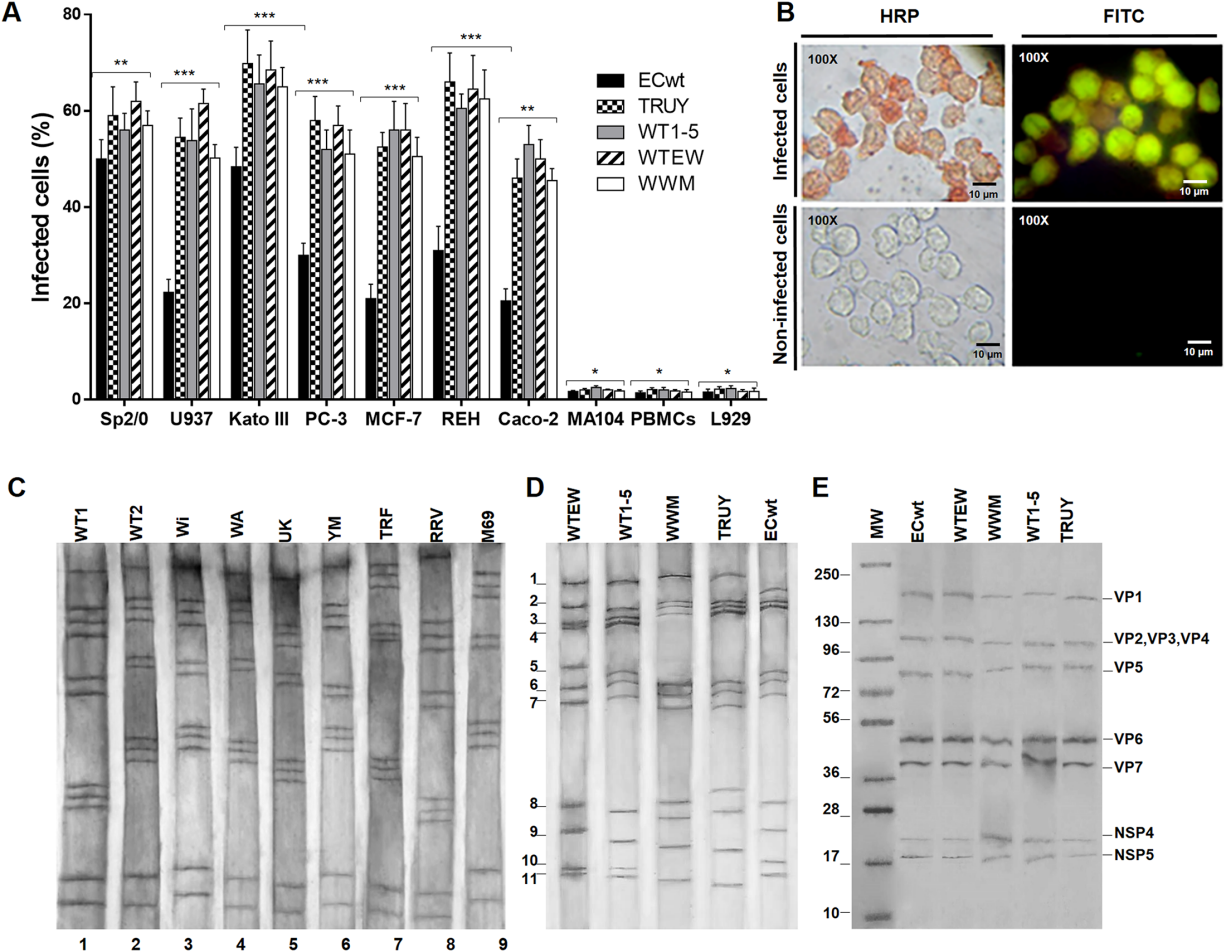
Rotavirus infection of tumor cell lines and electropherotyping of tumor cell-adapted rotavirus isolates WTWEW, TRUY, WT1-5, WWM and ECwt. **(A)** Tumor and non-tumor cell lines were separately infected with tumor-adapted rotavirus isolates WTWEW, TRUY, WT1-5, WWM or ECwt (MOI of 0.04) and harvested for analysis at 12 h.p.i. Uninfected cells were used as a control. Peripheral blood mononuclear cells (PBMCs) were used as non-tumoral control cells. Cells positive to rotavirus structural proteins (SP) were identified using immunochemistry and immunofluorescence assays. Asterisks represent statistically significant differences (***P< 0.001; **P< 0.01). Immunocytochemistry results are shown. **(B)** Representative images of immunochemistry (HRP) and immunofluorescence (FITC) assays are shown in cell samples taken at 12 h.p.i. **(C)** and **(D)** Rotavirus infected cells (MOI of 0.8) were collected at 24 h.p.i. and the viral dsRNA extracted with phenol-chloroform from cell lysates or cesium chloride purified virions. Electropherotyping was performed using SDS-PAGE (8%) and silver staining. Electropherotypes of parental rotavirus strains and isolates are indicated **(C)** Electropherotypes of the adapted/selected isolates are indicated **(D)** Lysates from infected cells with the indicated adapted/selected rotavirus isolates were analyzed by SDS-PAGE/Western blotting using antibodies against rotavirus SP and non-structural proteins NSP4 and NSP5.

Regarding that tumor cell-adapted may gain relative infectious capacity through mutation and/or reassortment, we wanted to test whether the combined rotaviruses (S1 Table) that are able to mutate and reassort gained higher infectious capacity than rotavirus ECwt that in principle is excluded from reassortment. To estimate the growth of the rotavirus isolate ECwt and the combined rotavirus isolates WT1-5, TRUY, WWM or WTEW in tumor cells Sp2/0-Ag14, U937, Kato III, MCF-7, REH and Caco-2, and in non-tumor cells MA104, L929 and PBMCs, the cells were inoculated with each isolate at a MOI of 0.04 as determined in MA104 cells. The rotavirus antigen accumulated in individual cells at 12 h.p.i. was determined by immunocytochemistry and immunofluorescence. The immunochemistry detection of viral antigen using antibodies against rotavirus structural and non-structural proteins (NSP4 and NSP5) revealed that all the combined isolates adapted to tumor cells were able to produce viral antigen-positive signals and their infectivity ranged from approximately 43% to 66% in the cells tested (Fig 1A). The infectivity of the tumor cell-adapted isolate ECwt was the lowest as compared to the tumor cell-adapted combined isolates. However, the infectivity in the tumor cells for all combined rotavirus isolates and ECwt isolate was significantly higher than that observed in the MA104 cells (about 5%), L929 cells or PBMCs (about 3%) (Fig 1A). The ECwt infectivity ranged from approximately 20% to 50% (Fig 1A). The results suggest that combined rotaviruses that can vary through mutation and reassortment seem to potentially gain higher infectious capacity than the non-combined isolates such as ECwt. Fig 1B shows a representative illustration of the correspondence between the immunochemistry and immunofluorescence detection of rotavirus antigens.

Eletropherotyping has been commonly used for characterizing rotaviruses [46] and some attempts have been made to correlate electropherotypes and serotypes [47]. A characteristic of rotavirus is its ability to reassort the genomic segments during co-infections [48]. To determine whether the rotavirus isolate ECwt, combined rotavirus isolates (WT1-5, TRUY, WWM or WTEW), parental rotavirus strains (Wa, M69, Wi, TRF, RRV, UK and Ym) or wild type isolates (WT1, WT2, WT3, WT4 and WT5) were able to induce in the infected cells the accumulation of the rotavirus dsRNA genomic segments and specific structural and non-structural NSP4 and NSP5 proteins, the infected cells were harvested at 24 h.p.i. and submitted to RNA and protein extraction. We wanted also to determine whether the gain-of-function experiments that boosted the infectious properties of rotavirus combinations following the multiple passages are related with a particular electropherotype. We used electropherotypes in order to differentiate the original parental strains or isolates from the finally tumor cell-adapted isolates. The results show that the resultant electropherotypes are neither the sum of the parental electropherotypes nor the selection of a particular parental electropherotype (Fig 1C and 1D). The eletropherotyping analysis suggests that the shift of the phenotypic characteristics of rotavirus combinations associated with gaining higher infectious capacity in tumor cells has been caused at least by the occurrence of reassortment. The presence of only 11 genomic segments in all the selected isolates suggests that they correspond to unique reassorted isolates rather than co-infections. However, mutations and remaining co-infections cannot be excluded whereas nucleotide sequencing of the selected genomic segments is not performed. After SDS-PAGE/Western blotting analysis of the phenol-extracted proteins, a typical pattern for rotavirus structural proteins was identified and any of the strains or isolates examined showed a differential protein pattern (Fig 1E). These results are indicative of rotavirus replication in the tumor cells tested.

**Fig 2:**
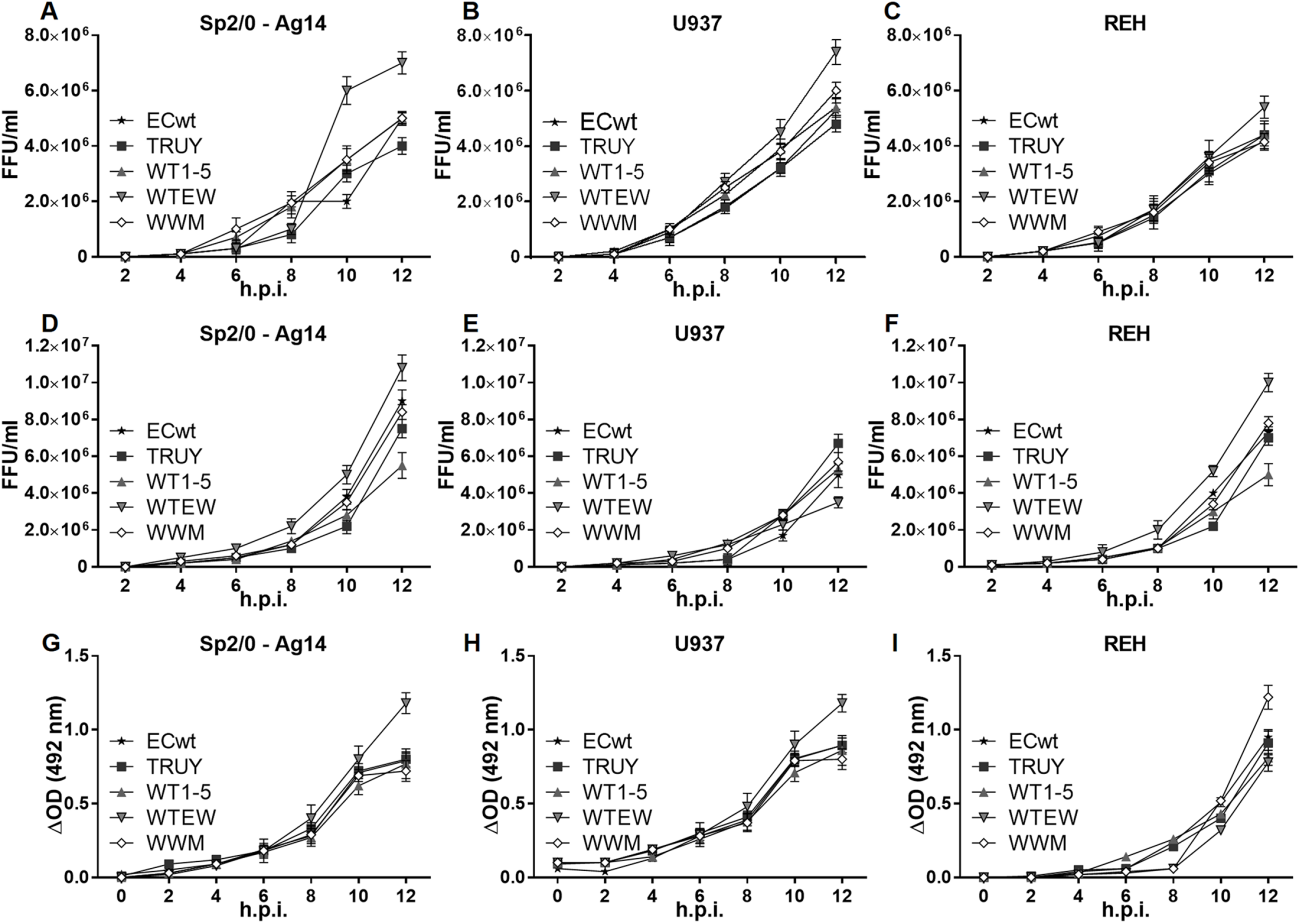
Time-course accumulation of infectious virions from tumor cell-adapted rotavirus isolates WTEW, TRUY, WT1-5, WWM and ECwt and accumulation of their viral structural proteins (SP). Titers of the rotavirus isolates indicated were determined using serial dilutions of infected cell lysates which were inoculated in the respective cell lines indicated. Titers are expressed in FFU/ml in the y-axis. Comparisons were performed by determining the dilution producing the infection of 50% of cells at 12 h.p.i. as determined by immunocytochemistry assay using antibodies against SP. The time-course accumulation of virus titer in cells Sp2/O-Ag14 **(A)** U937 **(B)** and REH **(C)** was followed by taking samples every 2 h until 12 h.p.i. for immunochemistry assay. Aliquots of the infected cells taken at the indicated post-infection times for **A**, **B** and **C** were frozen and thawed twice, and the supernatant of 700 *g* was activated with trypsin (10 μg/ml) to be inoculated in new cells Sp2/O-Ag14 (**D**) U937 (**E**) and REH (**F**). After 12 h.p.i., the cells were analyzed for the production of infectious titer determined by the presence of rotavirus SP using immunochemistry assay. Statistically significant differences for infectious titers between ECwt and the combined rotavirus isolates are described in the Results section. The activated supernatant from infected cell lines Sp2/O-Ag14 **(G)** U937 **(H)** and REH **(I)** was also examined for the presence of viral SP at the indicated post-infection times using capture ELISA.

To test whether the infected tumor cells Sp2/0-Ag14, U937 and REH were able to produce infectious virions, the cells were infected with rotavirus isolates WT1-5, TRUY, WWM, WTEW or ECwt at a MOI of 0.8 as determined in the respective tumor cell line. The cells were separately inoculated with each isolate and harvested every 2 h until 12 h.p.i. The profile of infectious particle increase was determined by assaying cell lysates from the indicated postinfection time points in new tumor cells Sp2/0-Ag14, U937 and REH. The immunochemistry assay showed that all tumor cell-adapted rotavirus isolates were able to produce increasing amounts of infectious virus particles through the postinfection time examined (Fig 2A–2C). Infectious particles in the cell lysates were present as early as 6–8 h.p.i. (Fig 2D–2F), which suggest that the final step of the viral cycle, assembly of new rotavirus virions, begun in average at this post-infection time. Infectious titers were significantly higher for all the combined isolates as compared with those of ECwt, particularly at 10 and/or 12 h.p.i. in Sp2/0-Ag14 cells (Fig 2A), whereas only WTEW and WWM exhibited higher infectious titers in Sp2/0-Ag14 cells at the last post-infection times (Fig 2B). Except WTEW at 12 h.p.i., any of the remaining combined isolates produced significantly different infectious titers in comparison with ECwt in REH cells (Fig 2C).

The production of infectious virions was further examined by infecting tumor cells as indicated immediately above and harvesting cells every 2 h until 12 h.p.i. The harvested cells were subjected to two cycles of freezing and thawing and centrifuged at 700 *g* for 10 min. The trypsin-activated cell lysates were tested at serial dilutions in new Sp2/0-Ag14, U937 and REH cells to determine their infectivity at the end of 12 h.p.i. After the immunocytochemistry assay, the results showed that the inoculum taken every 2 h from a previous infectious cycle were able to produce increasing amounts of infectious virions at the end of 12 h.p.i. (Fig 2D–2F). These results suggest that the infected cells collected every 2 h from an a previous infectious cycle contained increasing amounts of infection virions that were able in turn to produce increasing amounts of infection particles at the end of 12 h.p.i. in a new infectious cycle (Fig 2D–2F). Except WWM, all the remaining combined isolates showed significantly higher infectious titers than ECwt in Sp2/0-Ag14 cells (Fig 2D). Only TRUY and WTEW were able to produce significantly higher infectious titers than ECwt in U937 cells (Fig 2E). Except WWM, all the remaining combined isolates produced significantly higher infectious titers than ECwt in REH cells at the last post-infection times (Fig 2F).

To assess the release of viral antigens to the culture medium, the tumor cells Sp2/0-Ag14, U937 or REH were infected with the rotavirus isolates described immediately above. After inoculation, the cells were collected every 2 h until 12 h.p.i., by centrifugation and the supernatant tested by the presence of rotavirus antigens. The ELISA results indicated that structural viral antigens started to be detected at 4 h.p.i (Fig 2G–2I), which suggest that the infection process is progressing as virion-associated antigens are released into culture medium prior to cell lysis [49]. Regarding that no concomitant infectivity assay was conducted, the viral antigen detected in the culture medium cannot be exclusively attributed to mature virions as free structural proteins can also be released into the culture medium. Except WTEW at the last post-infection time, any of the remaining combined isolated released significantly different amount of viral antigen into the culture medium when compared with ECwt in Sp2/0-Ag14 cells (Fig 2G). Only WTEW at 12 h.p.i. and WT1-5 at 10 h.p.i. showed significantly higher viral antigen released into the culture medium in comparison to ECwt in U937 cells (Fig 2H). Similarly, WTEW and WT1-5 released significantly different amount of viral antigen into the culture medium when compared with ECwt (Fig 2I).

**Fig 3:**
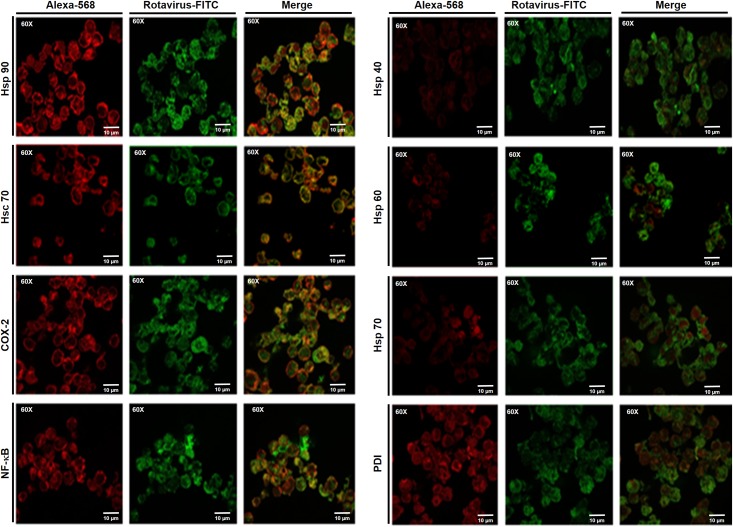
Co-localization of rotavirus structural antigens and cellular proteins HSP90, Hsc70, COX-2 and NF-κB in tumor cells. Sp2/0-Ag14 cells were infected at a MOI of 0.8 with tumor cell-adapted rotavirus isolates WTEW, TRUY, WT1-5, WWM or ECwt. Cells were harvested at 12 h.p.i., fixed with PFA and permeabilized. Co-localization was assessed using antibodies against viral SP (green) and cellular proteins Hsp90, Hsc70, Hsp60, Hsp40, Hsc70, PDI, COX-2 and NF-κB (red) using confocal microscopy. Merged images appearing yellow suggest co-localization.

Previous studies have shown that the cellular proteins Hsp90, Hsp70, Hsp60, Hsp40, Hsc70, NF-κB, COX-2 and PDI are altered in their expression as a consequence of rotavirus infection [45,50]. The tumor cells Sp2/0-Ag14, U937 or REH were infected with the rotavirus isolates WT1-5, TRUY, WWM, WTEW or ECwt as indicated above. After 12 h.p.i., the cells were fixed and permeabilized before treatment with antibodies to the rotavirus or cellular proteins previously indicated. Using confocal microscopy, it was found that the cellular proteins Hsp90, Hsp70, NF-κB and COX-2 co-localized with the viral antigens (Fig 3), suggesting that the viral proteins were intracellularly located as a product of the viral cycle.

### Cell viability of infected cells

**Fig 4:**
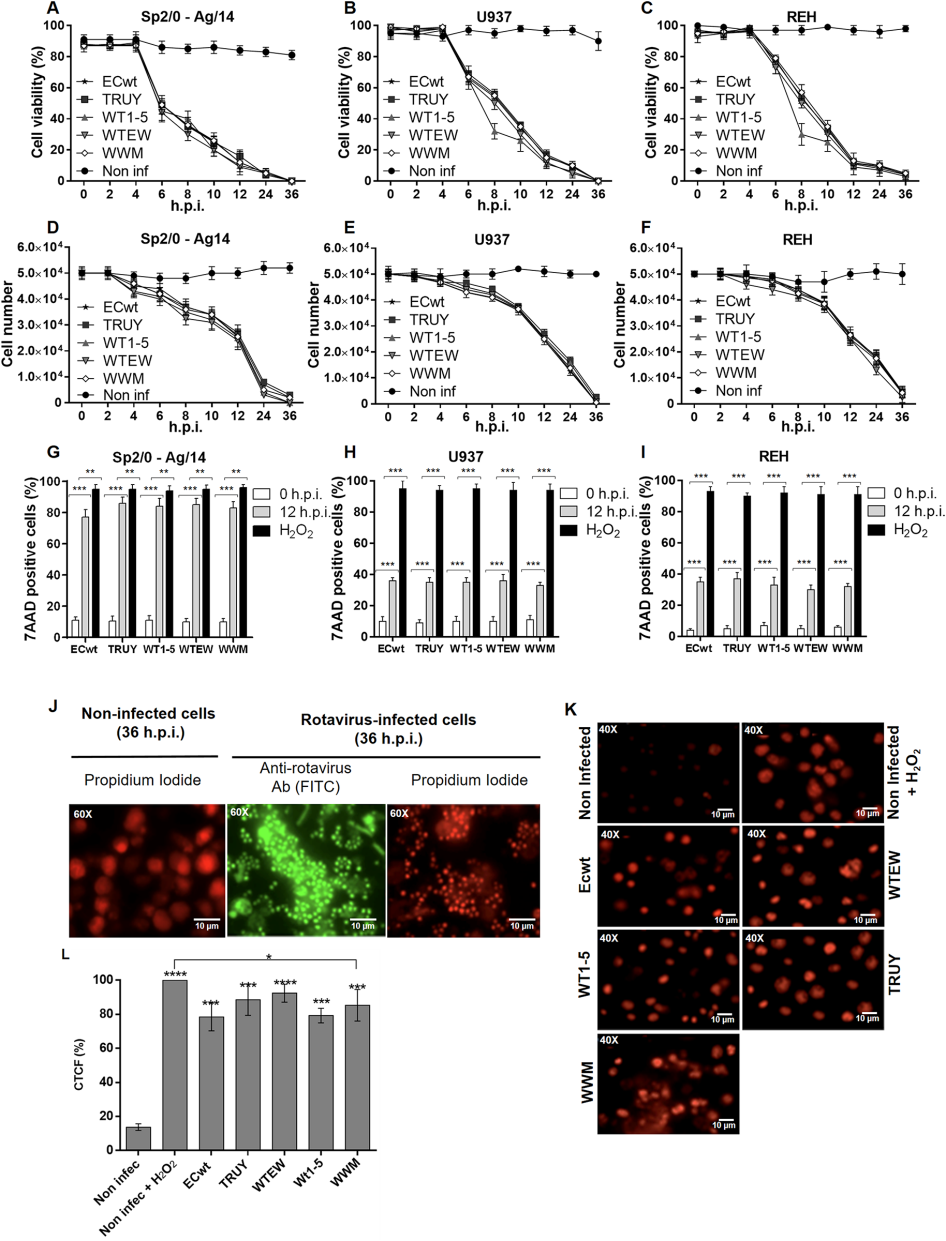
Decrease of cell viability and alterations of cell membrane permeability by infection with rotavirus isolates. The tumor cell lines indicated were infected with tumor cell-adapted rotavirus isolates WTEW, TRUY, WT1-5, WWM or ECwt at MOI of 0.5. Cell samples were collected every 2 h until 36 h.p.i. Viability of cells **(A)** Sp2/O-Ag14, **(B)** U937 and **(C)** REH, were determined using the trypan exclusion test. Total number of cells remaining after infection was determined for cells: **(D)** Sp2/O-Ag14, **(E)** U937 and **(F)** REH every 2 h until 36 h.p.i. using a Neubauer chamber. The absence of significant differences in the exponentially decaying profiles of cell viability and total cell population for all the viral isolates tested is described in Results section. Cell lines: **(G)** Sp2/O-Ag14, **(H)** U937 and **(I)** REH were infected with the rotavirus isolates (MOI of 0.8) indicated, collected at 12 h.p.i. and then tested for the integrity of their cell membrane using 7-AAD. Treatment with H_2_O_2_ was used as a control. Asterisks represent statistically significant differences (***P< 0.001; **P< 0.01). **(J)** Representative images of cell morphology of infected U937 cells examined at 36 h.p.i. using propidium iodide (red) and FITC labeled-antibodies against rotavirus SP (green) or **(K)** infected Sp2/0-Ag14 cells using 7-AAD. Fluorescence of 7-AAD positive cells shown in **(K)** was measured in randomly chosen triplicate fields per slide using the ImageJ program, and the corrected total cell fluorescence (CTCF) values in terms of percentage values are shown as mean (SD) in **(L)**. Asterisks indicate statistically significant differences (****P< 0.0001; ***P< 0.001; *P< 0.05).

Although mature rotavirus virions can be released into the medium before virus-induced cell lysis [49,51], the end of the viral cycle for many virus is associated with the lysis of the host cell. Some changes in cell membrane permeability occurring before cell death and lysis have been reported in rotavirus infected cells [52]. Cell viability in terms of cell membrane permeability changes for rotavirus infected-tumor cell lines was assessed using the Trypan blue exclusion test. Tumor cells Sp2/0-Ag14, U937 or REH were separately infected with rotavirus isolates WT1-5, TRUY, WWM, WTEW or ECwt at a MOI of 0.8 as indicated above. Cells were harvested every 2 h until 12 h.p.i. and then at 24 and 36 h.p.i. before treatment with Trypan blue solution. According to the proportion of cells internalizing the stain, it was observed that the cell viability began to decrease after 6 h.p.i. The mean percentage of viability for each cell line when it was separately infected with the five rotavirus isolates was 46.2%, 66.2% and 76.2% at this post-infection time for Sp2/0-Ag14, U937 and REH cells, respectively. The cell viability decreased to 12%, on average, for all cell lines by 12 h.p.i (Fig 4A–4C). The recording of the total number of cells during the same post-infection periods showed that, for all cell lines, this number decrease by 42–54% at 12 h.p.i. and 87–98.4% at 24 h.p.i. (Fig 4D–4F). Interestingly, the exponentially decaying profile of the cell viability percentage was concave whereas that of the total number of cells was convex. This result suggests that alterations of cell membrane occur rapidly after 4 h.p.i. whereas lysis leading to cell number decrease takes place in later stages of the infection process. No cells were observed at 36 h.p.i., except some cell debris that reacted with propidium iodide and antibodies to rotavirus (Fig 4J). However, the mechanisms explaining the profile of survival curves are still not well understood. For instance, many theoretical models have been proposed for explaining the convex (sigmoid) survival curve followed by mammalian cells [53,54]. Alterations of cell membrane prior to apoptotic signals and cell lysis have been reported for rotavirus-infected Caco-2 cells [1,51,55]. After comparing the specific exponentially decaying profiles of cell viability and total cell population for all the viral isolates tested, it can be concluded that on average no significant differences were observed for any of the viral isolates through the post-infection period examined in the cell lines indicated (Fig 4A–4F).

Assuming that as a cell dies its cell membrane becomes permeable to 7-AAD, tumor cells Sp2/0-Ag14, U937 or REH were infected with rotavirus isolates WT1-5, TRUY, WWM, WTEW or ECwt at a MOI of 0.8. The cells were treated with 7-AAD at 12 h.p.i. The percentage of cells being positive to the fluorescence was about 82.8%, 34.8% and 32% for Sp2/0-Ag14, U937 and REH cells, respectively (Fig 4G–4I and 4K). These percentages of death cells were lower than that observed in the control cells (about 100%) that had been treated with H_2_O_2_, whereas 3%, on average, of the uninfected and H_2_O_2_-untreated cells were positive to 7-AAD fluorescence (Fig 4K). The fluorescence of 7-AAD-positive cells was quantified using the program ImageJ. The corrected total cell fluorescence (CTCF) values showed that all the tumor cell-adapted isolates produced significant cell death effects relative to the uninfected control cells (Fig 4L). However, ECwt was slightly less effective in producing 7-AAD-positive cells than the combined isolates (Fig 4L).

### Cytopathic effects induced by rotavirus infection of tumor cells

**Fig 5:**
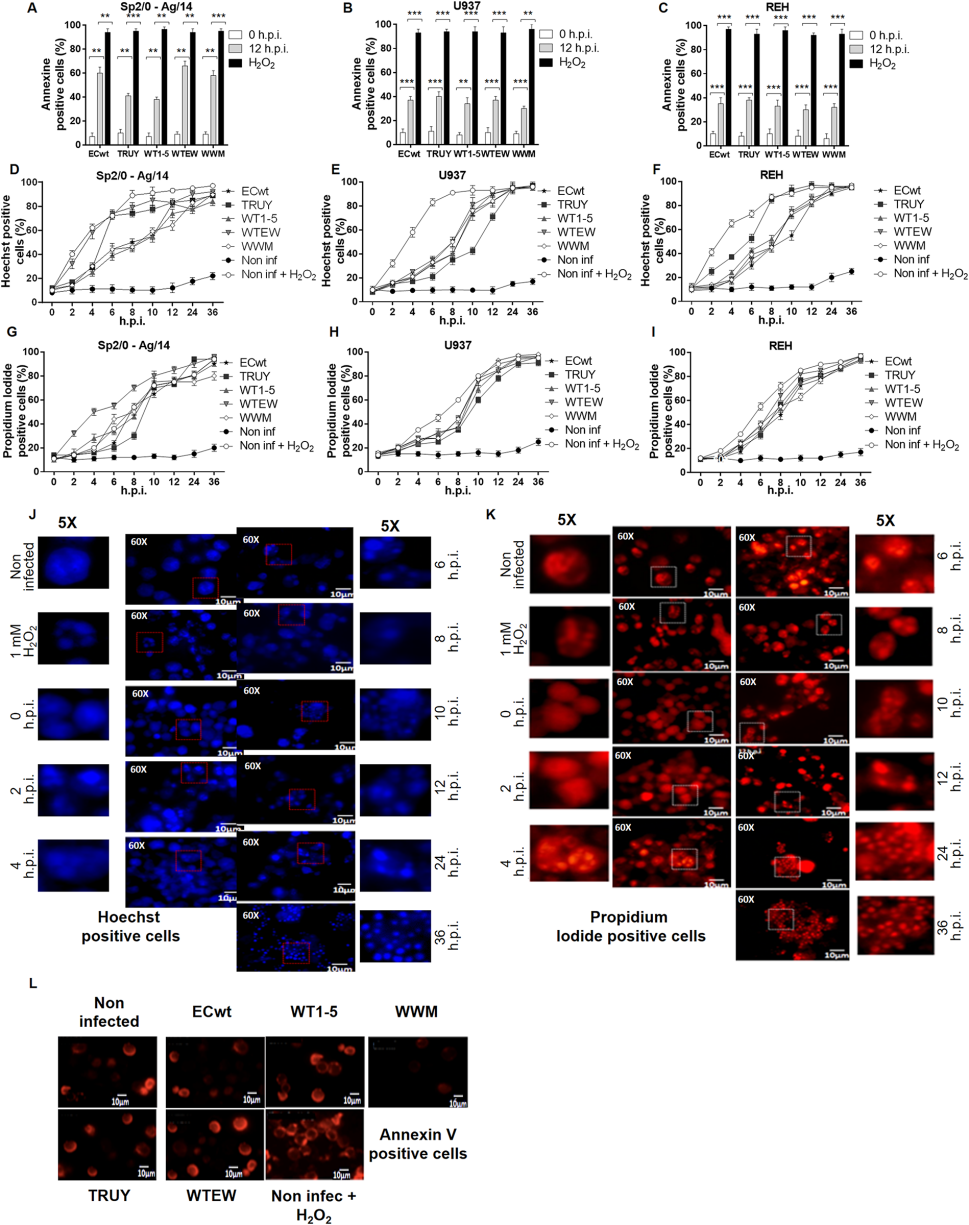
Phosphatidylserine flipping and nuclear morphology alterations induced by infection of rotavirus isolates. **(A)** Cell lines Sp2/O, **(B)** U937, and **(C)** REH were infected with tumor cell-adapted rotavirus isolates WTEW, TRUY, WT1-5, WWM or ECwt at MOI of 0.8. Annexin V expression was assessed at 12 h.p.i. H_2_O_2_ treatment was used as a control. Asterisks show statistically significant differences (***P< 0.001; **P< 0.01). **(D)** Cell lines Sp2/O, **(E)** U937 and **(F)** REH were infected as indicated for **(A)** through **(C)**, and then collected and fixed every 2 h until 12, 24 or 36 h.p.i. Chromatin condensation and nuclear fragmentation were determined using Hoechst. **(G)** Cell lines Sp2/O, **(H)** U937 and **(I)** REH were also examined with propidium iodide for their nuclear morphology. Statistically significant differences for the cytopathic effects induced by virus isolates are described in Results section. (**J)** Representative images of infected cell morphology examined with Hoechst, **(K)** propidium iodide and **(I)** Annexin V for cell line Sp2/O-Ag14 at different post-infection times.

To test whether the rotavirus infection was able to induce cytopathic effects on tumor cells Sp2/0-Ag14, U937and REH, these cells were separately infected with rotavirus isolates WT1-5, TRUY, WWM, WTEW or ECwt at a MOI of 0.8 as determined in the respective tumor cell. After addition of Annexin V-Alexa Fluor^®^ 568 to the cells at 12 h.p.i., the fluorescence microscopy analysis showed a fluorescence pattern that was similar to that observed in cells that had been treated with H_2_O_2_, except that the Annexin V- Alexa Fluor^®^ 568 positive signal was observed in a lesser proportion of rotavirus-infected cells (Fig 5A–5C and 5L). In fact, 67.5%, 59.5%, 49%, 41.5% and 36.5%, of Sp2/0-Ag14 cells were found to be positive to Annexin V- Alexa Fluor^®^ 568, when infected with rotavirus isolates WT1-5, TRUY, WWM, WTEW or ECwt, respectively, in comparison to H_2_O_2_-treated control cells (about 100%). U937 and REH cells were positive to Annexin V- Alexa Fluor^®^ 568 signal at percentages ranging from 31% to 36% (Fig 5A–5C and 5L). Taking into account the above-mentioned facts that virions were produced from 4 h.p.i., the viability started to decrease from 6 h.p.i., and the number of cells was decreased by about 50% at 12 h.p.i., the exact moment at which the Annexin V signal, indicative of an apoptotic effect, took place within the viral cycle cannot be exactly established.

To determine the chromatin condensation induced by rotavirus infection, infected cells were harvested every 2 h until 36 h.p.i. and stained with Hoechst 33342 or propidium iodide. The microscopic analysis of the stained cells with Hoechst (Fig 5D–5F and 5J) or propidium iodide (Fig 5G–5I and 5K) showed remarkable morphological changes in the nucleus such as a progressive chromatin condensation throughout the post-infection times examined. However some heterogeneity was observed among the different types of tumor cells and rotavirus isolates used for infection regarding the post-infection time at which the chromatin changes occurred and the proportion of cells showing these changes (Fig 5D–5K). For instance, only TRUY and WTEW induced significantly higher chromatin condensation than ECwt in Sp2/0-Ag14 cells before 10 h.p.i. (Fig 5D). Only TRUY at 10–12 h.p.i. showed significantly lower chromatin condensation than ECwt in U937 cells (Fig 5E). In contrast, TRUY induced significantly higher chromatin condensation than ECwt during 2–12 h.p.i. in REH cells (Fig 5F). When using propidium iodide instead of Hoechst 33342, only WTEW induced significantly higher chromatin condensation before 10 h.p.i. in comparison to ECwt in Sp2/0-Ag14 cells (Fig 5G). In U937 and REH cells, no significant differences in chromatin condensation were found between ECwt and the remaining isolates (Fig 5H and 5I).

**Fig 6:**
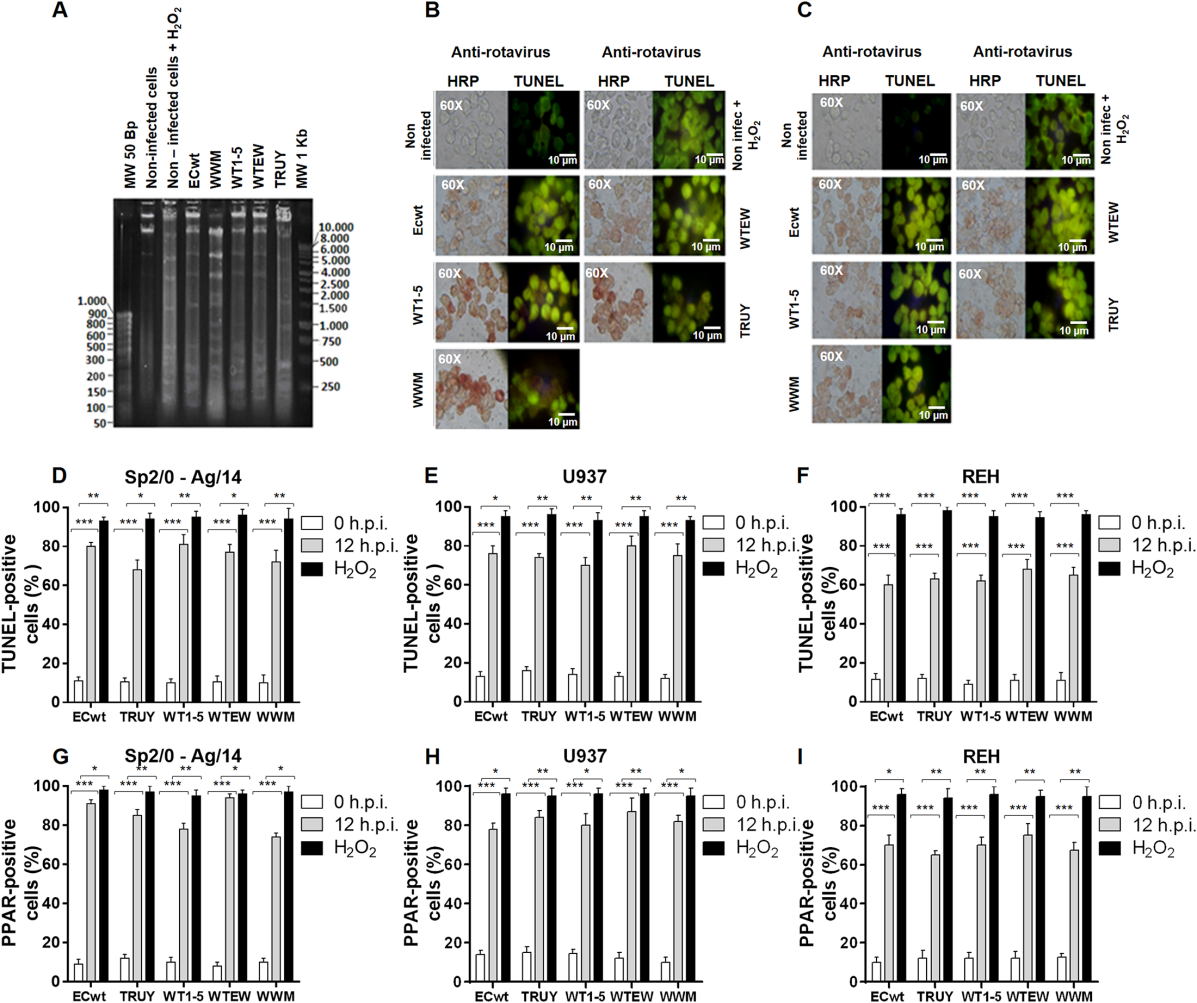
Nuclear fragmentation, DNA strand breaks and DNA repair induced by rotavirus infection. **(A)** Sp2/O-Ag14 cells were infected with tumor cell-adapted rotavirus isolates WTEW, TRUY, WT1-5, WWM or ECwt at a MOI of 2 and cells collected at 12 h.p.i. Phenol/chloroform extracted DNA was resolved in agarose gel (1%) electrophoresis and stained with ethidium bromide. H_2_O_2_-treated cells were used as a control. **(B)** Cells were infected with the virus indicated in **(A)** using a MOI of 0.8. Cells were examined for the presence of viral structural antigens using antibodies against SP in an immunochemistry assay (red) or cells were labeled for TUNEL and examined by immunofluorescence (green). **(C)** Cells were examined for the presence of viral antigens using antibodies against SP in an immunochemistry assay (red) or cells were incubated with antibodies against PARP (green). Uninfected cells and cells treated with H_2_O_2_ (1 mM) for 12 h.p.i. were used as a control. **(D, E and F)** and **(G, H and I)** The percentages of TUNEL- and PARP-positive cells, respectively, were determined by immunofluorescence assay. Asterisks show statistically significant differences (***P< 0.001; **P< 0.01; *P< 0.05). H_2_O_2_-treated cells were used as a control.

As programmed cell death involves degradation of DNA into high molecular weight fragments which are later degraded to low molecular weight fragments, the cleavage of DNA from tumor cells was studied after rotavirus infection (12 h.p.i.). The agarose electrophoretic analysis of DNA fragments from the tumor cells infected with the tumor cell-adapted rotavirus isolates showed a DNA fragmentation pattern that was similar to that observed in DNA from H_2_O_2_-treated control cells (Fig 6A). Most of DNA fragments were observed in the low molecular weight range of 300 bp or less. No differential patterns of DNA fragmentation were found in any of the cell types infected with the tumor cell-adapted rotavirus isolates studied (Fig 6A).

Tumor cells infected with the tumor cell-adapted rotavirus isolates were further analyzed at 12 h.p.i. for their DNA fragmentation pattern using a commercially available TUNEL kit. The same coverslips were also submitted to immunochemistry analysis for the presence of cell-associated rotavirus antigens. The TUNEL epifluorescence images of experimental cells were similar to those observed in control cells that have been treated with H_2_O_2_, whereas these images were in sharp contrast to those from uninfected control cells (Fig 6B). It deserves to be highlighted that TUNEL-positive cells correlated with cells being positive to rotavirus structural antigen (Fig 6B), which suggests at least an association between rotavirus infection and apoptotic effect. Regarding that during apoptosis PARP-1 is cleaved by caspase-3 into 24- and 89-kDa fragments, detection of this latter fragment can be used as an apoptosis marker [56,57]. Treatment of fixed cells with polyclonal antibodies to PARP-1 showed an intense fluorescent signal in the nucleus and cytoplasm of the rotavirus-infected cells. Similar but less intensive fluorescent images were found in the control cells treated with H_2_O_2_ (Fig 6C). As highlighted for TUNEL assay, cells being PARP-positive were also positive to rotavirus infection (Fig 6C).

The epifluorescence observation revealed that the percentage of the TUNEL-positive cells for cell lines Sp2/0-Ag14 and U937 reached more than 70%, whereas that for REH cells only surpassed 60% (Fig 6D–6F). As found for the TUNEL assay, more than 70% of Sp2/0-Ag14 and U937 cells were PARP-1-positive, whereas only more than 60% of REH cells showed fluorescent signals for PARP-1 (Fig 6G–6I). Taken together, these results indicate that rotavirus infection of the tumor cells studied is able to induce apoptotic signals leading to cell lysis.

**Fig 7:**
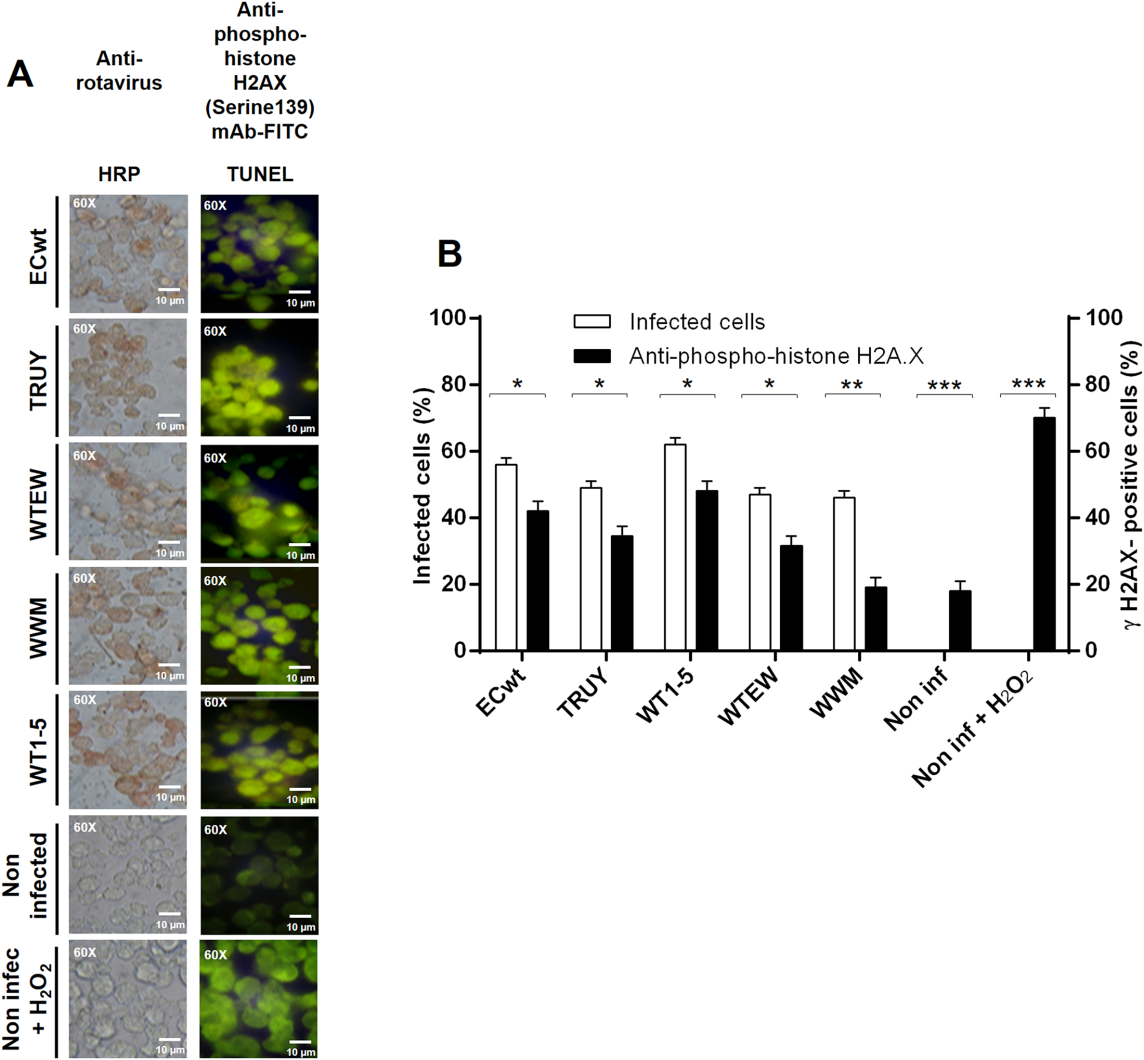
Induction of γH2AX accumulation by rotavirus infection. Sp2/O-Ag14 cells were infected with tumor cell-adapted rotavirus isolates WTEW, TRUY, WT1-5, WWM or ECwt at MOI of 0.8 and harvested at 12 h.p.i. **(A)** Immunocytochemistry analysis of infected cells using primary Abs against rotavirus SP and HRP-conjugated goat anti-rabbit secondary antibodies. For phospho-histone H2A.X analysis, cells were permeabilized with 0.5% Triton X-100 and blocked with 3% BSA in TBS buffer. Cells were treated first with anti-phospho-histone H2A.X (Ser 139) mAb (2 μg/ml) and then with FITC-conjugated goat anti-mouse secondary Ab (2 μg/ml). Uninfected cells treated or not with H_2_O_2_ (1 mM) were used as a control. **(B)** Comparison of the mean percentages of cells being positive to rotaviral antigen and γH2AX foci.

DNA double-strand breaks (DSBs) are able to induce a damage response (DDR) pathway that not only detect these lesions but also induces DNA repair or cell death if the lesions are too massive [58,59]. Histone H2AX is an important component of the DDR. H2AX phosphorylation status is indicative of DNA repair to survive or apoptosis to die [60]. Phosphorylation of histone H2AX at Ser139 to form γH2AX is induced in response to DNA DSBs. We found that the infection of Sp2/O-Ag14 cells with the five tumor cell-adapted rotavirus isolates led to an increased signal corresponding to the formation of γH2AX foci on DSB sites (Fig 7A). Quantification and comparison of the rotavirus antigen and the fluorescent intensity of the γH2AX signal showed that infection was associated with increased γH2AX foci (Fig 7B). Taken together, these results and those described above suggest that rotavirus infection of the tumor cell lines studied is able to induce apoptotic signals leading to cell lysis.

## Discussion

Cellular and host tropism represent one of the most relevant characteristics of viruses [61]. Virus replication needs a coherent virus-host cell interaction, which begins with the virion attachment to cell surface receptors previous to the entry into the cell and ends with the production of new virions [62]. Efficient oncolytic viruses depend on their tropism for neoplasic cells and lytic capacity to induce the death of tumor cells. In the present work we have adapted some rotavirus strains and isolates to efficiently infect and replicate in selected tumor cell lines and also resorted to experimental reassortment of some tumor cell-adapted rotaviruses. Reassortment has been found to modify the infectious capacity rotaviruses in wild type mice and mouse cells [63,64]. It deserves to be highlighted that all the reassorted isolates exhibited a significantly increased infectious capacity in the tumor cells studied in comparison to the parental rotavirus strains. This improved infectious capacity was evidenced by the accumulation of rotavirus-specific structural and non-structural proteins in the virus-infected tumor cells. Rotavirus replication in the tumor cells tested was also demonstrated by the accumulation of dsRNA genomic segments which exhibited electropherotype profiles that suggested they were derived by reassortment from the parental strains or isolates used during the initial co-infection of cells. However, the genetic identity of the reassorted genomic segments must be established after nucleotide sequencing. The time course analysis of the accumulation of infectious virions showed that the reassorted isolates were able to complete a whole viral cycle according to the profile of infectious titres. Although all the combined rotavirus isolates showed relatively higher percentages of viral antigen-positive cells than those of ECwt, However, that was not the case for their infectious titers and the virus infection-induced apoptotic signals as ECwt showed higher infectious titer and apoptotic effects than some combined isolates in some tumor cell lines. These differential virus effects depended on the type of combined isolate and/or the tumor cell line used. These findings suggest that tumor cell-adapted isolates differ in their specific virus-target cell interactions. These differences might be reflecting their genetic heterogeneity caused by mutation and/or reassortment occurring during their tumor cell adaptation.

We wanted to approach the mechanisms by which rotaviruses induce the death of tumor cells. Whether the cell death induced by rotavirus infection involves apoptosis [65], necrosis [66] or oncosis [67] has not been clearly established. We found that infection of tumor cells by the reassorted rotavirus isolates led to chromatin condensation and DNA fragmentation which suggest that apoptosis could be involved in the rotavirus-induced tumor cell death. According to DNA fragmentation, rotavirus-induced apoptosis has been suggested in cultured tumor cell lines such as human intestinal Caco-2 cells [54]. However, some studies have shown that apoptosis and cell death mechanisms induced by rotavirus infection depend on cell type and degree of differentiation [68,69]. The results shown in the present work suggest that the reassorted rotavirus isolates obtained by multiple passages of parental rotavirus in tumor cells were successful in attaching and entering to cells, avoiding the innate immune response and controlling the host translational machinery. Although rotaviruses are able to stimulate IFN-β and early antiviral gene expression, they also have mechanisms to avoid or suppress the INF effects associated with innate or adaptive [69,70]. Rotaviruses encode proteins, such as NSP1, that antagonize the IFN-β expression pathway through the induction of transcription factor degradation [64,71]. Rotaviruses, as other RNA viruses, have the ability of inducing changes to the host translational machinery in order to facilitate the synthesis of viral proteins and inhibit the synthesis of cellular proteins [72]. Rotavirus-induced inhibition of cellular mRNA translation is mediated by the activities of its nonstructural protein NSP3 [73].

Mixing rotaviruses isolated from different host species, followed by multiple passages in tumor cells, could have favored the emergence of novel reassortants highly pathogenic for tumor cells. However, the analysis of the infectious capacity of these novel isolates showed a wide variability in their infectivity. Regarding the cytopathic effects induced by these isolates in tumor cells, it should be highlighted that the first viral infectious particles were detected as early as 6 h.p.i. when an inoculum (MOI of 0.8) infecting about 50% of cells was used. At this post-infection time, the cell viability ranged from 45 to 70%, showing that rotavirus infection had significantly affected cell viability by the time of initiation of virion production. At 12 h.p.i., a post-infection time at which at least two viral cycles had occurred, the cell viability had decreased to 12%, on average, for all tumor cells studied, and the percentage of infected cells ranged from 43 to 66%. Given that the total number of tumor cells had decreased by about 50% at 12 h.p.i., it is quite plausible to suggest that cells undergoing the first viral cycle were completely lysed by this post-infection time.

The phospholipid asymmetry of the cell membrane is altered during early apoptosis leading to the exposure of phosphatidylserine on the outer layer of the cell membrane. Uninfected tumor cells were largely negative for Annexin V at 12 h.p.i., whereas 36 to 67% of Sp2/0-Ag14 cells infected with the reassortant rotavirus strains were Annexin V-positive depending on the reassortant rotavirus used (Fig 5A). However, only 31 to 36% of U937 and REH cells were found to be Annexin V-positive when the cells were infected with the different reassortants (Fig 5B and 5C). Percentages of 7-AAD-positive cells similar to those found with Annexin V assay were detected for the rotavirus-infected cells at 12 h.p.i., except that Sp2/0-Ag14 cells showed more dispersion as the percentage of 7-AAD-positives cells fluctuated widely around 80% for all reassorted isolates assayed (Fig 4G–4I). Taken together, cells being permeable to the nuclear dye 7-AAD and positive to Annexin V are supposed to be apoptotic cells. However, it should be highlighted that cell death induced by oncolytic viruses does not exactly follow the conventional patterns of apoptosis or necrosis but rather shows some characteristics that are specific for both kinds of cell death with some variations depending on the oncolytic virus type [74]. Phosphorilation of histone H2AX occurs in response to DSB formation resulting in γH2AX. Infection of tumor cells with rotavirus isolates resulted in a significant percentage of γ-H2AX-positive cells. It has been shown that γH2AX appears during apoptosis concomitantly with the appearance of high molecular weight DNA fragments [75]. γH2AX formation in rotavirus infected cells is indicative that infection was able to induce chromatin modifications that are compatible with apoptotic DNA fragmentation. Overall, the results reported here provide evidence about the adaption of five rotavirus isolates that were able to replicate and induce cell death in some tumoral cell lines. To the best of our knowledge, this is the first study showing that rotaviruses are able to infect and kill efficiently tumor cells. Although rotaviruses safety could be anticipated, further research must be conducted in order to make sure that rotaviruses meet the features characterizing an ideal oncolytic agent. In addition to direct oncolysis, rotaviruses should be able to induce immune-mediated anticancer effect and possible synergistic interactions with other cancer treatments without inducing antiviral adaptive immune response.

## Supporting Information

S1 Table Parental rotavirus strains or isolates and combined isolates. (DOCX)
