# Supplementary material for: Experimental Adaptation of Rotaviruses to Tumor Cell Lines
Source: PLoS One. 2016 Feb 1;11(2):e0147666. doi: 10.1371/journal.pone.0147666 (PMC4734670; doi:10.1371/journal.pone.0147666)
Supplement: S1 Table — (DOCX) [file pone.0147666.s001.docx]

**Supplementary Table 1**

**Table 1**. Parental rotavirus strains/isolates and combined isolates.

| **Combined isolates^a^** | **Parental strains^b^/isolates** |
| --- | --- |
| **WT1-5** | Five wild isolates from patient feces |
| **TRUY** | **T**RF (Porcine), **R**RV (simian), **U**K (Bovine), **Y**M (Porcine) |
| **WWM** | Human strains **W**a, **W**i, and **M**69 |
| **WTEW** | **W**T1-5, **T**RUY, **E**Cwt and **W**WM |

**^a,b^**All strains/isolates were passaged at least 100 – 150 times in tumor cell lines.
